# Sulphate of Phyllerine

**Published:** 1849-10

**Authors:** 


*Sulphate of Phyllerine.*—M. Jachelli, of Ferrara, has lately added this alkaloid to the list of febrifuges; it is obtained from the well known evergreen shrub, *Phyllerea Latifolia.* It was known, before the researches of Dr. Jachelli, as a cooling astringent, but it is now found to possess the same active anti-periodic properties as others of its class, the ash, the olive, etc.

An extensive series of experiments have been made since the year 1825, on the action of this alkaloid in agues, by Dr. Jachelli. He has compared its operation with that of—1st, a powder of the young leaves and twigs, in doses of 30 grains during the intermission; 2d, a simple decoction of the plant to 60 of water, down to one-third, and given in large doses also during the intervals; 3d, with a compound decoction formed by adding 30 minims of dilute sulphuric acid to the preceding. The sulphate, in doses of from 12 to 15 grains during the apyrexia, has evinced its superior activity over other preparations of the phyllerea; thus of 20 patients treated with the sulphate, 20 were cured; of 13 to whom the powder was administered, 11 were cured; of 18 to whom the compound decoction was given, 14 were cured; of 16 who took the simple decoction, 7 were cured.—*Bulletin General de Therapeutique.*

